# Ketorolac-Loaded PLGA-/PLA-Based Microparticles Stabilized by Hyaluronic Acid: Effects of Formulation Composition and Emulsification Technique on Particle Characteristics and Drug Release Behaviors

**DOI:** 10.3390/polym15020266

**Published:** 2023-01-04

**Authors:** Amaraporn Wongrakpanich, Nichakan Khunkitchai, Yanisa Achayawat, Jiraphong Suksiriworapong

**Affiliations:** 1 Department of Pharmacy, Faculty of Pharmacy, Mahidol University, Bangkok 10400, Thailand; 2 Doctor of Pharmacy Program, Faculty of Pharmacy, Mahidol University, Bangkok 10400, Thailand

**Keywords:** microparticle, ketorolac, PLGA, PLA, drug release, osteoarthritis

## Abstract

This study aimed to develop ketorolac microparticles stabilized by hyaluronic acid based on poly(lactide-co-glycolide) (PLGA), poly(lactide) (PLA), and their blend for further application in osteoarthritis. The polymer blend may provide tailored drug release and improved physicochemical characteristics. The microparticles were prepared by water-in-oil-in-water (w/o/w) double emulsion solvent evaporation using two emulsification techniques, probe sonication (PS) and high-speed stirring (HSS), to obtain the microparticles in different size ranges. The results revealed that the polymer composition and emulsification technique influenced the ketorolac microparticle characteristics. The PS technique provided significantly at least 20 times smaller average size (1.3–2.2 µm) and broader size distribution (1.5–8.5) than HSS (45.5–67.4 µm and 1.0–1.4, respectively). The encapsulation efficiency was influenced by the polymer composition and the emulsification technique, especially in the PLA microparticles. The DSC and XRD results suggested that the drug was compatible with and molecularly dissolved in the polymer matrix. Furthermore, most of the drug molecules existed in an amorphous form, and some in any crystalline form. All of the microparticles had biphasic drug release composed of the burst release within the first 2 h and the sustained release over 35 days. The obtained microparticles showed promise for further use in the treatment of osteoarthritis.

## 1. Introduction

Microparticles have generally been developed and extensively applied to treat various diseases. It is of interest to fabricate biodegradable polymeric microparticles since numerous compounds, including peptides, pharmaceutical proteins, and hydrophobic medicines, can be encapsulated in the microparticles and administered into the body. Customizing the physicochemical properties of polymers enables control of the release characteristics of such delivery systems, which can be varied over the desirable period from days to months. Poly(lactide-co-glycolide) (PLGA) and poly(lactide) (PLA) are the most frequently utilized for the fabrication of delivery systems, including microparticles, thanks to their biodegradability, biocompatibility, various unique characteristics, and human use approval by USFDA [1]. PLGA or PLA microparticles are typically created using various methods, for example, emulsification solvent extraction/evaporation, spray drying, and microfluidic [2]. Emulsification solvent evaporation has been extensively employed to create microparticles after the solidification of particle core by solvent evaporation. Numerous techniques, such as high-speed stirring, and ultrasonication, can be used to emulsify and generate tiny oil droplets. Depending on the formulation and process setting, microparticles emulsified by high-speed homogenization or ultrasonication often have a wide range of particle sizes and microstructural morphology [3]. Diffusion, particle erosion, or a combination of these processes play a significant role in the release of drugs from biodegradable polymeric microparticles. Different size microparticles have illustrated unique characteristics of drug encapsulation and release [4].

Osteoarthritis (OA) is a chronic degenerative disorder characterized by low-grade systemic inflammation and the degeneration of joint-related tissues such as articular cartilage [5]. OA produces joint pain, which is often exacerbated by weight-bearing and activity, as well as stiffness following inactivity [6,7]. Weight control, medication, and supportive therapies are the most common treatments for OA. In some cases, intra-articular injection therapies or surgery may be required. Ketorolac tromethamine is one of the non-steroidal anti-inflammatory drugs (NSAIDs) used to treat OA. The drug solution can be administered via intraarticular injection (IA), which produces equipotent pain relief and functional improvement to IA corticosteroids [8,9]. Unlike corticosteroids, injecting ketorolac intraarticularly shows no sign of cartilage damage. The major issue of ketorolac is a short half-life in the body; hence more frequent administration is required leading to patient non-compliance. In addition, hyaluronic acid can also be co-administered with ketorolac, which enables a more rapid analgesic onset with no serious complications [10,11]. Moreover, injecting hyaluronic acid along with ketorolac can enhance joint space narrowing and bone marrow density [12].

Ketorolac microparticles have been widely investigated using different types of polymeric materials and preparation methods. Polymethacrylate (Eudragit^®^) microparticles for oral administration were fabricated by oil-in-oil solvent evaporation [13]. This method yielded high encapsulation efficiency with a particle size range of 75–225 µm. Parenteral ketorolac-loaded albumin microspheres were also produced by the emulsion cross-linking method. A wide range of encapsulation efficiency from 21–59% was obtained [14]. Other ketorolac microparticles have also been studied based on ethyl cellulose [15], Carbopol, polycarbophil, chitosan [16], PLGA, PLA, poly(ε-caprolactone) (PCL) [17,18,19] as well as the blend of two polymers such as chitosan/gelatin [20]. To regulate the physicochemical properties of polymers, a blend of at least two polymers has been extensively utilized. Despite the fact that polymer blends are rarely miscible, they may produce new materials or combinations with enhanced performance. On the contrary, immiscible blends can provide materials with phase separation in the microstructure. Eventually, the physicochemical characteristics of the materials rely on the degree of compatibility between the blend components. A few reports have produced the ketorolac microparticles from PLGA, PLA, and their blends with PCL [17,18,19]. The encapsulation efficiency of ketorolac was reported to be dependent on blending content, the inherent viscosity of the polymer, the lactide ratio in PLGA polymer, and particle size. Higher encapsulation efficiency was obtained when the formulation was composed of pure PLGA or PLA, a higher ratio of lactide, more inherent viscosity, and larger particle size [17,18]. Moreover, the blends of PCL with PLGA or PLA provided sustained release characters depending on the PLGA or PLA contents. Increasing PLGA or PLA ratios retarded the drug release from a few days to a few months [17,18]. Nonetheless, the PLGA/PLA blend microparticles for encapsulating ketorolac have not been reported. PLGA and PLA blends may provide better physicochemical behaviors of materials and lead to a customized degradation rate, modifiable drug release, and better mechanical characteristics.

Therefore, this study aimed to develop ketorolac microparticles based on PLGA, PLA, and their blend to deliver ketorolac in the presence of hyaluronic acid. As previously described, hyaluronic acid can be co-administered with ketorolac, and the developed formulations are intended to be administered intraarticularly in alleviating osteoarthritis. A key determinant in the quality of microparticles prepared by the emulsification evaporation method is a surfactant or stabilizer. In this study, hyaluronic acid was used as a stabilizer in water phase 1 and concurrently used with polyvinyl alcohol (PVA), a typical stabilizer for the double emulsion method [21], in a water phase 2 to ensure the formation of double emulsion droplets. Two techniques, probe sonication and high-speed stirring, were employed for the water-in-oil-in-water (w/o/w) emulsification to render two size ranges of the microparticles. Different compositions and emulsification techniques may provide unique properties of the microparticles. To the best of our knowledge, comparisons of different emulsification techniques and microparticle size ranges have rarely been reported. Furthermore, no published study has reported ketorolac-loaded PLGA/PLA microparticles and PLGA- and PLA-based microparticles formulating with hyaluronic acid and PVA as stabilizers. Hence, the characteristics of the prepared microparticles were evaluated in terms of size, size distribution, morphology, encapsulation efficiency, yield, thermal behaviors, crystallinity nature, and release behaviors.

## 2. Materials and Methods

### 2.1. Materials

Poly(DL-lactide-co-glycolide) (PLGA, 50:50, inherent viscosity 0.59 dL/g, MW 53.4 kDa) and poly(L-lactide) (PLA, inherent viscosity 1.16 dL/g, MW 158.0 kDa) were purchased from Durect Corporation, Birmingham, UK. Ketorolac (MW 255.27 g/mol) in the form of ketorolac tromethamine (water solubility 200 mg/mL, log P 2.1 [22]) was obtained from Cayman Chemical Company, Ann Arbor, MI, USA. Hyaluronic acid (300–500 kDa, Nanjing Gemsen International Co. ltd, Nanjing, China), polyvinyl alcohol (PVA, Mowiol^®^ 8-88, MW 67 kDa, Sigma-Aldrich Pte. Ltd., Singapore), acetone (Honeywell Burdick & Jackson, Morris Plains, NJ, USA), dichloromethane (DCM, Honeywell Burdick & Jackson, Morris Plains, NJ, USA), methanol (high-performance liquid chromatography (HPLC) grade, Honeywell Burdick & Jackson, Muskegon, MI, USA), and sterile water for irrigation (SWI, General Hospital Products. Public Co., Ltd., Pathum Thani, Thailand) were used as received.

### 2.2. Microparticle Preparation

The microparticles were prepared using the w/o/w double emulsification solvent evaporation method using two emulsification techniques, namely probe sonication and high-speed stirrer, to obtain the different sizes of the microparticles. Three different formulations were prepared by both techniques, resulting in 6 individual formulations.

#### 2.2.1. Probe Sonication (PS) Technique

A water phase 1, 0.8 mL, containing 1% *w*/*v* hyaluronic acid with or without 50 mg of ketorolac was emulsified in an oil phase containing 200 mg of polymer dissolved in 2 mL of DCM by probe sonicator (Vibra-Cell Processors VCX 130, Sonics & Materials, Inc., Newtown, UK) at 40% amplitude for 60 s. The primary emulsion was further emulsified in 8 mL of water phase 2 containing 0.375% *w*/*v* PVA and 1% *w*/*v* hyaluronic acid (pH 3.0) by a probe sonicator at 40% amplitude, 60 s. The w/o/w emulsion was then poured into 7 mL of 1% *w*/*v* hyaluronic acid (pH 3.0) under continuous stirring, and then the solvent was evaporated under reduced pressure. The microparticles were collected and subjected to lyophilization (Crist Alpha 1–4 freeze dryer, SciQuip, Newtown, UK) for 72 h. The lyophilized microparticles were kept at −20 °C overnight for further study.

#### 2.2.2. High-Speed Stirring (HSS) Technique

The primary emulsion was prepared using a similar protocol as the PS technique. After obtaining the primary emulsion, the w/o/w emulsion was prepared by mixing the primary emulsion with 8 mL of water phase 2 using Ultra-Turrax^®^ homogenizer (T 25 digital, IKA^®^, Staufen im Breisgau, Germany) at a speed of 4000 rpm for 1 min. Then, it was mixed with 7 mL of 1% *w*/*v* hyaluronic acid (pH 3.0) by a magnetic stirrer in a fume hood for the particles to solidify. The microparticles were obtained after lyophilization for 72 h. The lyophilized microparticles were kept at −20 °C overnight for further study.

### 2.3. Characterization of Particles

#### 2.3.1. Particle Size and Size Distribution

The particle size (d (0.5) and d (0.9)) and size distribution (span) were measured using Mastersizer 2000E (Malvern Instrument Ltd., Malvern, UK). The sample was prepared by dispersing the dry microparticles in SWI at a concentration of 20 mg/mL. During the measurement, the sample was diluted in reverse osmosis water. The measurement was performed in triplicate.

#### 2.3.2. Zeta Potential

The zeta potential of the particles was determined by Zetasizer NanoZS ((Malvern Instrument Ltd., Malvern, UK). The sample was prepared using the same method for particle size measurement. The measurement was performed in triplicate.

#### 2.3.3. Drug Loading Content

The drug loading (%DL) and entrapment efficiency (%EE) of ketorolac-loaded microparticles was measured as follows. A known amount of lyophilized microparticles was degraded in 0.3 N sodium hydroxide and sonicated for 15 min. The sample’s pH was adjusted to 7.0 with diluted hydrochloric acid, and the volume of the sample was adjusted to 5 mL with 50% *v*/*v* methanol in SWI. The sample was filtered and diluted before HPLC analysis. The %DL and %EE were computed according to Equations (1) and (2), respectively.

(1) $$\%\mathrm{DL}=\frac{\mathrm{Analyzed}\;\mathrm{amount}\;\mathrm{of}\;\mathrm{drug}}{\mathrm{Weight}\;\mathrm{of}\;\mathrm{sample}}\times 100$$

(2) $$\%\mathrm{EE}=\frac{\mathrm{Analyzed}\;\mathrm{amount}\;\mathrm{of}\;\mathrm{drug}}{\mathrm{Initial}\;\mathrm{weight}\;\mathrm{of}\;\mathrm{drug}}\times 100$$

The validated HPLC analysis was performed using an isocratic mode, Agilent 1200 series HPLC instrument (Agilent Technologies Inc., Santa Clara, CA, USA) according to the United State Pharmacopoeia with some modifications [23]. The drug was eluted through ACE^®^ C18-1104 reverse phase column (150 × 4.6 mm, 5 µm, Advanced Chromatography Technologies Ltd., Scotland, UK) with a guard column. The mixture of acetic acid in water (1:44 *v*/*v*) and methanol (40:60% *v*/*v*) at a flow rate of 1 mL/min was used as a mobile phase. The drug was detected by a diode array detector at a wavelength of 265 nm.

#### 2.3.4. Yield

After the lyophilization of the microparticles, the processing yield of the microparticles was determined compared to the initial amount of solid content used in each formulation.

#### 2.3.5. Morphology Observation

The surface morphology of the microparticles was visualized using a field-emission scanning electron microscope (FESEM, Schottky FESEM JSM-7610F, JEOL Ltd., Tokyo, Japan). The sample was fixed on a glass slide mounted on the SEM stub and coated with platinum. The SEM images were captured using an emission current of 2.0 kV at magnifications of 10,000×–20,000×.

### 2.4. Differential Scanning Calorimetry

The thermal behaviors of the formulations were evaluated using a differential scanning calorimeter (DSC 3+, Mettler Toledo Limited, Greifensee, Switzerland). The sample was placed in an aluminum pan with a lid. The measurement was performed under nitrogen flow. The sample was heated from room temperature to 200 °C (1st heating), then cooled down to 20 °C and re-heated to 200 °C (2nd heating) at a temperature rate of 20 °C/min. The diffractogram of the 2nd heating was analyzed for enthalpy of heating (ΔH), the onset of endothermic peak (T_m_), and the glass transition temperature (T_g_).

### 2.5. X-ray Diffraction

The crystallinity of the drug in the microparticle formulations was analyzed by an X-ray diffraction instrument (Miniflex, Rigaku Americas Holding Company, Inc., Wilmington, MA, USA). The dry sample was placed on a glass slide. The measurement was conducted at two-theta (2*θ*) in the range of 0°–60° and a rate of 0.06 degree/s.

### 2.6. In Vitro Release Study

The release of ketorolac from the formulations was investigated using the dialysis method in a simulated physiological fluid pH 7.4. Briefly, the freshly lyophilized microparticles were reconstituted in SWI. Two milliliters of the microparticles were put into a dialysis bag (MWCO 6–8 kDa, CelluSep^®^T2 Membrane Filtration Products, Inc., Seguin, TX, USA) and immersed in 20 mL of phosphate-buffered saline pH 7.4 containing 0.02% sodium azide as a preservative. The study was conducted at 37 °C and 100 rpm for 5 weeks. At each time point, 1 mL of the release fluid was taken, and an equal volume of fresh warm medium was replaced immediately. The sample was analyzed by HPLC as previously described.

### 2.7. Statistical Analysis

The data are expressed as mean±SD from at least three measurements. One-way ANOVA or Student’s *t*-test was used to compare the means of multiple groups or two groups, respectively. The difference was considered to be significant if the *p*-value < 0.05. The release profiles were compared using repeated measures two-way ANOVA to compare the percent cumulative drug release at any time and the release profiles of different formulations [24].

## 3. Results

### 3.1. Physicochemical Properties of Microparticles Prepared by Different Emulsification Techniques

In this study, we aimed to fabricate two different size series of microparticles by the w/o/w emulsification–solvent evaporation method using different emulsification techniques, namely PS and HSS. Each series was composed of three formulations using different compositions of PLGA and PLA, namely, 100% PLGA, 50% PLGA:50% PLA, and 100% PLA. PLA exhibits higher hydrophobicity than PLGA. The different polymer compositions can affect the physicochemical properties and release behaviors of the drug-loaded microparticles. The critical step in microparticle preparation is the emulsification of w/o primary emulsion, which may affect the stability of the emulsion and the capacity of drug encapsulation. To avoid any issue in this critical step, we prepared w/o primary emulsion by probe sonication for both techniques. Then, the w/o/w secondary emulsion was prepared by different emulsification techniques. Hyaluronic acid (1%) was used as a stabilizer in water phases 1 and 2; however, a small amount of PVA at a final concentration of 0.2% *w*/*v* was also added in water phase 2 as an auxiliary stabilizer. In our preliminary study (data not shown), water phase 2 without pH adjustment produced very low drug encapsulation efficiency. It has been reported that the low encapsulation efficiency of ketorolac is attributed to the high water solubility of ketorolac tromethamine. This leads to the drug partition into continuous water phase 2 during emulsification [18,25]. Since ketorolac is a weak acid (pKa 3.5) [26], the solubility can be reduced in an acid-dispersing phase. The acidified external phase increases the encapsulation efficiency of ketorolac [17]. Therefore, in this study, water phase 2 was adjusted to pH 3.0 before the emulsification process, while water phase 1 was used without pH adjustment.

All of the blank microparticles prepared using the PS technique had a median size (d (0.5)) of 3.12–7.12 µm while the HSS technique yielded a significantly larger microparticle size with the range of 56.32–97.44 µm (Table S1, *p*-value < 0.05). All of the blank microparticles had negative zeta potential (ZP) ranging from −3.8 mV to −9.8 mV. The PS technique yielded a broader size distribution than the HSS technique (*p*-value < 0.05). Therefore, these two techniques could produce microparticles with different desired size ranges.

Three formulations (PLGA, PLGA/PLA, and PLA) of ketorolac-loaded microparticles were prepared using both PS and HSS techniques. The results are summarized in Table 1. After encapsulation, all of the formulations had significantly smaller particle sizes (d (0.5) and d (0.9)) (*p*-value < 0.05) when compared to the blank microparticles. At the same time, their span value was insignificantly different (*p*-value > 0.05), suggesting no effect of the drug on the size distribution of the microparticles. The zeta potential of most drug-loaded formulations became less negative than the blank ones, attributable to the presence of the drug on the microparticle surface.

To investigate the effect of the polymer composition on the microparticle characteristics, three different polymer compositions of drug-loaded microparticles were prepared. PLA exerts higher hydrophobicity than PLGA. The blending of PLGA and PLA at a 1:1 mass ratio would modify the hydrophilic–hydrophobic balance of the individual polymers. It was found that PLA microparticles had the smallest d (0.5), d (0.9), and span values among all formulations prepared by the PS technique. Meanwhile, the blending of PLGA and PLA resulted in the largest d (0.9) and span values. Interestingly, when preparing by the HSS technique, the PLGA/PLA and PLA microparticles possessed comparable d (0.5) and d (0.9), and the smallest particles were obtained from PLGA microparticles.

All of the microparticles prepared by the PS technique had at least 20 times smaller sizes than those by HSS. The PS technique yielded smaller d (0.5) and d (0.9) but wider size distribution than the HSS technique (*p*-value < 0.05). These two techniques basically have different principles. Probe sonication generates a physical vibration and a high shear force by ultrasonic sound waves from the tip of the probe [27]. Differences in the sound intensity (compression–rarefaction cycles) or the sound wave will affect the efficiency of emulsion droplet size reduction and particle deagglomeration [28]. The shear force decreased with the distance far from the probe; thus, the emulsification may not be uniformly distributed throughout the emulsion mixture resulting in wide size distribution. Nevertheless, HSS facilitates emulsification by consistent shear force. The emulsion mixture is forced through a shaft space and consistently shear the emulsion into small droplets. This technique generally provides uniform shear force throughout the sample and thus yields the uniform size of the particles. The bigger microparticles did not result from the technique but from the designed low-speed homogenizer in order to prepare the microparticles in different sizes. Increasing the Ultra-Turrax^®^ homogenizer speed or increasing the homogenization time, which exerts higher energy density and shear stress into the system, could reduce the particle size.

In addition to the particle emulsification technique, other factors can affect the particle size and size distribution, such as the viscosity of the internal phase. The higher the viscosity of the internal phase, the more difficult it was to break down into small droplets. PLA used in this study had higher molecular weight and inherent viscosity than PLGA. Thus, it was anticipated that the PLA microparticles would be larger than the PLGA microparticles. This phenomenon occurred in large microparticles. The microparticles made of PLA (HSS) and PLGA/PLA (HSS) showed larger particle sizes than the microparticles made of PLGA (HSS). However, all of the particles fabricated using the PS technique had small sizes in the same range (1–2 μm). The PS technique is potentially more powerful than the HSS and generates enough energy to break down the emulsion into very small droplets.

The formulation compositions had an insignificant effect on %yield, %DL and %EE (*p*-value > 0.05). This occurred in both particles prepared by PS and HSS techniques. However, there was a correlation between %EE and the polymer composition. For the PS technique, %EE tended to increase with the PLA component. On the other hand, the drug encapsulation efficiency by HSS was lower in the pure PLA formulation. One of the factors determining the quality of the microparticles manufactured by solvent evaporation is the rate of solvent evaporation [29]. A higher evaporation rate limits the rate of drug diffusion and reduces drug losses. Additionally, it has been established that the viscosity of the oil phase and the size of emulsified oil droplets affect the solvent evaporation rate and, thus, the microparticle encapsulation efficiency. Despite PLA having the highest molecular weight and inherent viscosity among other formulations, the PLA (PS) microparticles exhibited the highest %encapsulation efficiency among all of the PS formulations. This was possibly attributed to the more predominant effect of particle size than the viscosity of the oil phase. The very small size of the PLA particles had the largest surface area and, thus, the highest solvent evaporation rate preventing drug leakage during preparation [18]. On the contrary, the PLA microparticles produced by HSS had the lowest %EE compared to other HSS formulations due to the lowest surface area of the particles and the highest viscosity of PLA, slowing down the evaporation of the solvent and thus allowing drug loss during solvent evaporation. This result is consistent with the previous reports [18,29]. Different emulsification methods showed no effect on %yield, %DL, and %EE, except for the PLA microparticles. The %EE of the PLA (PS) formulation was significantly greater than that of the PLA (HSS) microparticles. The PS technique generated smaller droplets with a larger surface area, allowing for a higher solvent evaporation rate. Finally, it limited drug leakage and improved encapsulation efficiency, as previously described.

In conclusion, the emulsification technique clearly affected the particle size and size distribution. The encapsulation efficiency of ketorolac was influenced by the polymer composition and the emulsification technique. The molecular weight and the intrinsic viscosity of the polymers, as well as the solvent evaporation rate, play important roles in the entrapment efficiency of the big microparticles. On the other hand, the solvent evaporation rate insignificantly impacts the entrapment efficiency of the small microparticles with a comparable size range.

**Figure 1:**
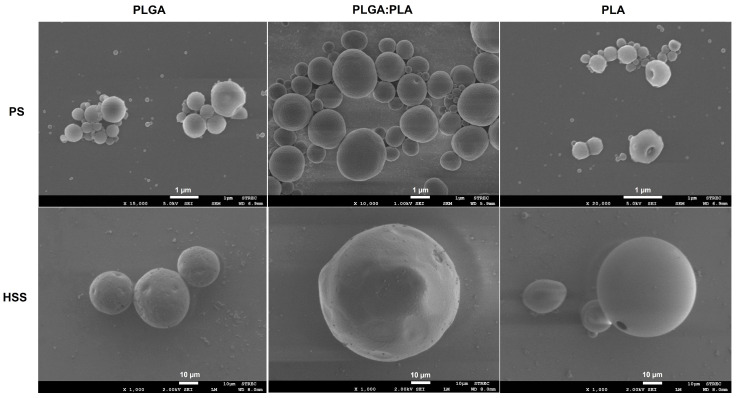
FE-SEM images of ketorolac-loaded microparticles. Scale bars in PS and HSS techniques represent 1 μm and 10 μm, respectively.

The morphology of the ketorolac-loaded microparticles was visualized by FESEM, as shown in Figure 1. Clearly, the microparticles fabricated using the PS technique were smaller than HSS-fabricating microparticles. Regardless of the emulsification techniques, all particles had an almost spherical shape. Overall, the PLA formulations had smoother surfaces than the PLGA formulations, although some PLA particles had pores, possibly due to the sample preparation.

### 3.2. Differential Scanning Calorimetry (DSC)

**Figure 2:**
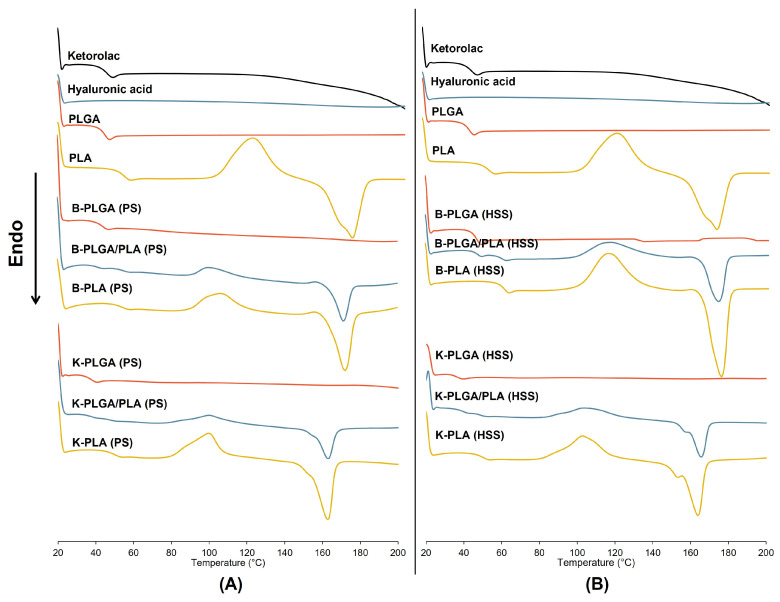
Differential scanning calorimetry thermograms of blank (B-PLGA, B-PLGA/PLA, and B-PLA) and ketorolac-loaded microparticles (K-PLGA, K-PLGA/PLA, and K-PLA) prepared by PS (**A**) and HSS (**B**) techniques.

The thermal behaviors of the microparticles were analyzed to study the possible interaction and polymorphism of the components. The DSC thermograms are illustrated in Figure 2, and the thermal parameters are summarized in Table 2. It has been reported that PLGA and PLA have unique characteristics depending on their molecular weight and composition [1]. Our results revealed that PLGA had a T_g_ at 42.8 °C without any endothermic peak, suggesting its amorphous nature. This is in agreement with the literature that PLGAs containing less than 70% glycolide are amorphous [30]. Meanwhile, PLA had a T_g_ at 53.3 °C, a double cold crystallization exotherm (T_cc_) at 110 °C and 121 °C and a recrystallization melting endothermic peak at 173 °C (156–180 °C) and an enthalpy of 45.5 J/g, suggesting the semicrystalline nature of the polymer. The cold crystallization of PLA is attributed to the nucleation of the melt state when heating from the glassy state [31,32].

The T_g_s of PLA and PLGA of the blank microparticles slightly changed from the polymers (Table 2). HSS increased the T_g_s of PLGA and PLA, whereas PS had a slight impact on the glass transition of the polymers. The blended polymers of the PLA/PLGA microparticles had a minimal change of glass transition, suggesting the partial miscibility of PLGA and PLA in the blend. All of the PLA-containing microparticles had lower T_cc_, ΔH_cc_, and ΔH_m_ compared to those of the pure PLA polymer, while their T_m_ of PLA was retained. A single cold crystallization peak was detected in the PLA and PLGA/PLA formulations except in PLA (PS) formulation, suggesting that the cold crystallization of PLA-containing microparticles occurred through heterogeneous nucleation [31]. The T_cc_ of PLGA/PLA microparticles further decreased compared to the PLA microparticles because of the nucleating effect by the PLGA phase and PLA crystalline domains. The microparticles prepared by HSS had higher T_cc_, ΔH_cc_, T_m_, and ΔH_m_ than the PS technique. Thus, both emulsification techniques affected the thermal behaviors due to the energy applied to break down the droplets during the preparation. PS generated higher energy, while HSS employed lower mechanical energy to break up the droplets prior to the solidification step. Moreover, the smaller droplet size by PS had a much higher surface area than those by HSS for solvent removal, leading to more rapid solidification of the particle core and a shorter time for polymer chain rearrangement. Thus, the PS technique had a greater impact on the thermal behaviors of the polymers than HSS.

In the case of drug-loaded microparticles, the presence of the drug dramatically reduced the T_g_s of PLGA and PLA by 4–7 °C and 3–4 °C, respectively (Table 2). A decrease in the T_g_ of all of the drug-loaded microparticles compared to the blank formulations and the pure materials may suggest the compatibility of the drug and the polymer. The drug was molecularly dissolved in the polymer matrix due to the plasticizing effect [4,33]. Compared to the blank microparticles prepared by the PS technique, the drug-loaded microparticles had almost unchanged T_cc_, increased ΔH_cc_ and ΔH_m_, and decreased T_m_. By HSS, the drug-loaded microparticles had lower T_cc_, ΔH_cc_, T_m_, and ΔH_m_ than the blank microparticles. Interestingly, all of the ketorolac-loaded PLA-containing microparticles had double cold crystallization and melting events, suggesting that the encapsulation of the drug interfered with the cold crystallization of the polymer, leading to heterogeneous and homogeneous nucleation [34,35]. The physical mixture of the blank PLA microparticles and ketorolac shows similar crystallization patterns and melting events in the thermograms to the blank PLA microparticles, except that the double melting peak was observed. Ketorolac showed only T_g_ without an endothermic peak in the second heating; however, it had two melting peaks at 161 and 168 °C in the first heating cycle (Figure S1), suggesting the crystalline nature of the drug, which turned to amorphous after rapid cooling. The double melting event of the drug-loaded PLA microparticles was possibly attributed to either the presence of the crystalline state of the drug or the thicker heterogeneous lamellar layers of the crystals. Therefore, we further investigated the crystallinity of the drug in the microparticle samples by XRD.

### 3.3. X-ray Diffractometry (XRD)

**Figure 3:**
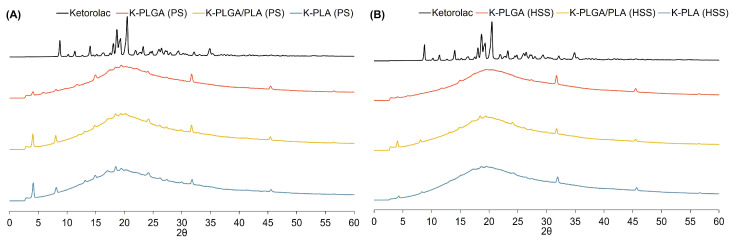
X-ray diffractograms of ketorolac-loaded microparticles (K-PLGA, K-PLGA/PLA, and K-PLA) prepared by PS (**A**) and HSS (**B**) methods.

The crystallinity of the drug-loaded microparticles was investigated by powder XRD. As shown in Figure 3, major characteristic peaks of ketorolac tromethamine appeared at 8.76°, 13.98°, 18.06°, 18.66°, 19.32°, and 20.52° confirming the crystalline nature of the drug. The diffractograms of all ketorolac-loaded microparticles showed that the intensity of these peaks evidently reduced. This result suggested that the majority of the drug was in an amorphous form and coexisted with some crystalline form, possibly due to an unencapsulated drug. The peaks at 31.68° and 45.48° belonged to hyaluronic acid and appeared in all diffractograms of all microparticles.

### 3.4. In Vitro Drug Release Study

**Figure 4:**
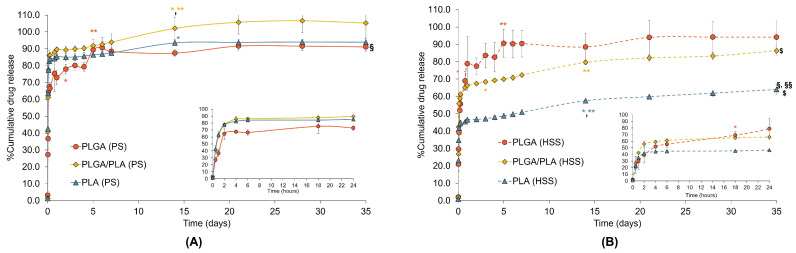
The cumulative release profiles of ketorolac from ketorolac-loaded microparticles prepared by PS (**A**) and HSS (**B**) methods in PBS, pH 7.4, over 35 days (mean ± SD, n = 3). An inset represents the release profiles within 24 h. Solid lines and dotted lines represent the drug release from the microparticles prepared by PS and HSS techniques, respectively. *, ** Significantly different when comparing %cumulative drug release at the time forward with 4 and 24 h, respectively; ^§^ significantly different when comparing the release profile with that of PLGA/PLA microparticles prepared by the same technique; ^§§^ significantly different when comparing the release profile with that of PLGA microparticles prepared by the same technique; ^$^ significantly different when comparing the release profiles of the similar component microparticles prepared by PS and HSS techniques.

The release of ketorolac from different formulations was studied in phosphate-buffered saline (PBS) pH 7.4 using the dialysis method. In the previous reports [8,9,36], ketorolac was intraarticularly administered once a week for 5 weeks. Therefore, our release study was conducted for 35 days. The release profiles are illustrated in Figure 4. All microparticles exhibited biphasic release consisting of the initial rapid release phase followed by the sustained release of the drug over 35 days. The initial drug release from all PS microparticles by 64–77% was extremely fast within the first 2 h (Figure 4A). Meanwhile, only 39–55% of the drug was released from the HSS microparticles, followed by the slow release of the drug with different release rates, depending on the formulations, until the end of the study (Figure 4B). The difference in the initial release rate of PS and HSS microparticles may primarily be due to their different size and surface area. It has been stated that the drug release of PLGA microparticles is known to be principally influenced by a number of variables, including particle size, porosity, and polymer molecular weight [37]. Many reports have demonstrated that particle size is a primary determinant of drug release rate [4,38,39]. The release of drugs from the different sizes of PLGA microparticles was affected by the rates of swelling and water penetration of the particles, mainly related to the surface area [40]. The smaller PLGA microparticles (4–40 µm) had a burst release of 20% within the first day owing to their faster swelling rate than the larger particles (40–125 µm). Another study demonstrated that the small size range microparticles (<20 and 20–50 µm) had rapid and complete drug release within the first week, while the larger size microparticles (50–100 and >100 µm) showed slow release within the first week [4]. The initial fast release of the ketorolac-loaded PS microparticles may be attributed to the rapid rates of swelling and water penetration entailing matrix porosity and permeability of the microparticles. In addition to the water penetration rate, the drug adsorbing on the surface of the microparticles and the lyophilization process can affect the initial release. It is believed that the initial rapid release is associated with the release of drug molecules trapped close to the microparticle surface and a great initial drug concentration gradient between the particles and the aqueous medium [41]. During lyophilization, the drug migration may cause a heterogeneous drug distribution in the polymer matrix and result in rapid or burst release [42]. The changes in pore size, geometry, and pore interconnectivity during the freezing and lyophilization process may also contribute to this issue [43]. In the case of the HSS microparticles, the initial burst release contributed to the heterogeneous size distribution in the formulations. The PLGA, PLGA/PLA, and PLA (HSS) microparticles contained d(0.1) of 17.5, 36.1, and 15.9 µm, meaning that 10% of the samples had a size below the mentioned value. The concomitant presentation of small particles released the drug more quickly and increased the burst effect [37]. Our results are in agreement with the previous report [4]. The unfractionated microparticles exhibited the release pattern combining those of small and large microparticles. The burst release mainly originated from the smaller microparticles, while the sustained release phase arose from the large microparticles.

Comparing the different emulsification techniques when using the same formulations, the release profiles of the ketorolac-loaded PLGA microparticles prepared by both techniques were comparable over 35 days (*p*-value > 0.05). Nevertheless, both formulations had significantly different initial drug releases within the first 4 h (*p*-value < 0.05). The PLGA (PS) microparticles had a faster drug release of 67.5 ± 1.5% than the PLGA (HSS) microparticles (52.0 ± 9.1%) due to the smaller size and faster rate of water penetration as previously described. After 4 h of drug release, both formulations exhibited indifferent release profiles (*p*-value > 0.05). Both formulations had comparable T_g_ values below 37 °C, which was the studied temperature. It is possible that the glass transition of the particle core had a greater impact on the drug release from the PLGA microparticles than their particle size. Considering the microparticles made from the PLGA/PLA and PLA polymers, the smaller PS microparticles enabled significantly faster drug releases than the larger HSS microparticles (*p*-value < 0.05) due to a higher surface area and a greater rate of water penetration, as aforementioned.

Comparing the different formulations prepared by the same method, the drug release from the PLGA/PLA microparticles in the PS series was tentatively faster than in the PLA microparticles. The PLGA microparticles yielded the slowest drug release. In this study, PLGA has higher hydrophilicity and smaller molecular weight than PLA. Based on the properties of the polymers at a similar size of microparticles, the blended PLGA/PLA should have the drug release slower than pure PLGA but quicker than pure PLA. However, the result was inconsistent with the hypothesis, possibly due to polydisperse size distribution, thermal behavior, and the partial compatibility of drug and polymer. The PLGA/PLA microparticles had various sizes (1–19 µm) with a high span value of 8.50 ± 2.83. The small size fraction may be responsible for the fast release of the drug in the heterogeneous size distribution sample. Furthermore, faster drug release from PLGA/PLA microparticles was also possibly attributed to their lower T_g_ than the studied temperature (from PLGA blended, 35.85 °C) and the partial compatibility between polymer and drug (from PLA blended). At the studied temperature (37 °C), higher than T_g_, the particle core transformed to a rubbery state, leading to faster drug diffusion and release. The ΔC_p_ (specific heat change) in the glass transition region of PLGA/PLA microparticles (0.151 and 0.101 J/g for PLGA and PLA regions, respectively) was lower than that of PLGA microparticles (0.211 J/g), suggesting a smaller free volume when transitioning to a rubbery state and thus a minimal volume for the drug [33]. In addition, blending PLGA with PLA reduced the compatibility between drug and polymers, as evidenced by two T_g_s of the PLGA/PLA blend in the thermograms. Thus, these circumstances contributed to a more rapid release of the drug from the PLGA/PLA microparticles than the PLGA and the PLA formulations. Despite the fact that the PLGA microparticles had a larger size than the PLA microparticles, the PLGA microparticles showed the slowest drug release. The compatibility of the PLGA with ketorolac, the water-soluble drug, was more critical than T_g_ and particle size. The higher drug release of the PLA microparticles than the PLGA microparticles contributed to the incompatibility of ketorolac with PLA and the smallest size of the particles.

In the HSS series, the PLA microparticles released the drug slower than the PLGA and PLGA/PLA microparticles (*p*-value < 0.05). The particle size and hydrophilic/hydrophobic nature of the polymers play an important role in the drug release profile of large microparticles. The PLGA microparticles had a smaller size and higher hydrophilicity than the others, resulting in a higher rate of water uptake into the particles. In addition, they also had a T_g_ lower than 37 °C. Thus, these factors made the polymer matrix more permeable and facilitated the drug release from the microparticles. On the other hand, the PLA microparticles showed the slowest and lowest drug release (*p*-value < 0.05). The slow release of the drug began after the initial 4 h at a constant rate. At the end of the 5-week study, only 64.1 ± 3.2% of the drug was released from the PLA microparticles. We further studied the drug release of this formulation for additional 3 weeks. The release of ketorolac gradually reached 81.6 ± 6.2% by the end of week 8 (data not shown). Their sustained release pattern was possibly attributable to the higher crystalline nature, larger molecular weight, and more hydrophobicity of PLA compared to the PLGA/PLA blend and the pure PLGA. Therefore, these factors hindered water penetration, polymer matrix permeability, drug diffusion, and drug release from the particle core [37].

In addition to the effects of the particle size and the hydrophilic/hydrophobic nature of the polymers on the drug release profiles of the HSS microparticles, other possible factors affecting the release profiles of ketorolac are pore closing, polymer degradation, and particle erosion [37,44,45,46]. The pore closing of the PLGA microparticles occurred during the incubation of the microparticles in a PBS medium at 37 °C [44]. This effect was more predominant with increasing temperature, while it was unlikely to be observed at a lower temperature. The pore closing effect was attributed to the flexibility of the polymers at the studied temperature. If the polymer has a T_g_ nearby or lower than the studied temperature, the polymer chains possess the flexibility for pore closing. Consequently, the drug release was suddenly changed to a slower rate. In our study, the microparticles of PLGA, with a comparable MW with the previous report [44], exhibited a T_g_ value lower than 37 °C, at which the pore closing may occur upon incubating the particles in the aqueous release medium. However, the blending of PLGA and PLA in the PLGA/PLA microparticles led to the partial miscibility of the matrix core and may limit the chain flexibility of PLGA. Thus, the polymer chains of PLGA in the blend may become less flexible than the PLGA microparticles while having more flexibility than the PLA microparticles for pore closing. On the other hand, the pore closing effect was less pronounced in the case of the PLA microparticles since they possessed a T_g_ value much higher than 37 °C, which limits the flexibility of the polymer chain during incubation. Regarding polymer degradation and erosion, it is well-known that the drug release from PLGA or PLA microparticles is governed by drug diffusion, polymer degradation, and polymer erosion [37,40,46,47]. Both polymers undergo mainly hydrolysis of the ester linkages [46], but their degradation and erosion rates are considerably different depending on polymer crystallinity, lactide-to-glycolide mole ratio, polymer molecular weight, water absorption, and T_g_ [37,45,46]. It is believed that water hydration in the matrix of an amorphous PLGA is greater than that of semicrystalline PLA, and thus, PLGA is more accessible to water and susceptible to hydrolysis than the homopolymer PLA [46]. The polymer degradation process normally begins much earlier than the polymer erosion. In the previous report, PLGA degradation occurred in a few days, while its erosion took a few weeks [40,46,47]. Our formulations contained different compositions of the polymers. So, this factor may contribute to the different release profiles of ketorolac from the HSS microparticles since they were much larger than the PS microparticles. The drug release of PLGA microparticles was the highest, followed by the PLGA/PLA and PLA microparticles. Thus, it was postulated that the matrix of PLGA formulations might be degraded faster than the PLGA/PLA and PLA formulations, respectively. Although our study has not investigated the degradation and erosion of the particles, it was hypothesized that the microparticles might be degraded or eroded during the release study. Nonetheless, further studies are required to prove these hypotheses.

According to the previously reported clinical trials [8,9,36], ketorolac and hyaluronic acid were concomitantly given once a week by intraarticular injection for 5 weeks. It is known that ketorolac has a short half-life of 4–6 h, and the use of the drug is needed frequent administration. Based on the study design of ketorolac and hyaluronic acid in OA patients, the developed microparticles could be employed in the patients once a month since the release of ketorolac could be retarded for 35 days. The sustained release of ketorolac could maintain the drug in articular fluid for a desirable period. From our results mentioned above, the fast release PS microparticles can be combined with the sustained release HSS formulations to achieve the therapeutic level as fast as the first day of injection and for the whole treatment period. The PLA (PS) microparticles, having a fast release of ketorolac within 24 h and narrow size distribution, can be a choice for combining with any HSS formulations, depending on the desired extent and rate of drug release.

## 4. Conclusions

This study demonstrated the effects of polymer composition and emulsification technique on the ketorolac-loaded microparticles. The ketorolac-loaded PLGA-/PLA-based microparticles with different size ranges were successfully prepared by PS and HSS techniques. Our study was designed based on the intraarticular injection of ketorolac and hyaluronic acid once a week continuously for 5 weeks in OA patients. The PS microparticles exhibited higher drug release within 24 h, while the HSS microparticles demonstrated the sustained release of ketorolac over 35 days. The combination of fast release PS microparticles and sustained release HSS formulations can be used once a month as an alternative regimen in OA patients, which may enhance patient compliance and minimize drug usage and administration costs. The obtained microparticles demonstrated potentiality for the treatment of OA. However, an efficacy investigation of these combinations in animals and patients is required before further application.

## Figures and Tables

**Table 1:** Characteristics of ketorolac-loaded microparticles.

Formulation	Particle Size (µm)		Span	Zeta Potential (mV)	%Yield	%Drug Loading	%Entrapment Efficiency
	d (0.5) *	d (0.9) *					
PLGA (PS)	2.16 ± 0.00	5.89 ± 0.01	2.20 ± 0.01	−3.9 ± 0.1	49.98 ± 8.59	8.83 ± 0.65	76.32 ± 5.58
PLGA/PLA (PS)	2.12 ± 0.11	19.20 ± 6.70	8.50 ± 2.83	−3.5 ± 0.4	55.56 ± 4.13	9.24 ± 1.32	79.83 ± 11.38
PLA (PS)	1.36 ± 0.01	3.08 ± 0.19	1.47 ± 0.13	−3.5 ± 0.1	55.83 ± 7.08	9.45 ± 0.34	81.63 ± 2.94
PLGA (HSS)	45.50 ± 0.06	64.76 ± 0.15	1.04 ± 0.00	−4.4 ± 0.3	46.79 ± 6.81	8.93 ± 2.15	77.15 ± 18.61
PLGA/PLA (HSS)	67.43 ± 0.40	101.32 ± 1.32	0.97 ± 0.01	−3.8 ± 0.2	58.18 ± 5.47	8.60 ± 0.37	74.28 ± 3.20
PLA (HSS)	60.88 ± 0.54	103.51 ± 1.67	1.44 ± 0.01	−3.6 ± 0.2	50.29 ± 3.16	7.82 ± 0.56	67.52 ± 4.84

* d (0.5) and d (0.9) mean the size below which 50% and 90% of the sample are contained, respectively.

**Table 2:** Temperatures of glass transition (T_g_), cold crystallization (T_cc_), and melting (T_m_) and enthalpies of cold crystallization (ΔH_cc_) and melting (ΔH_m_) of individual components, blank and drug-loaded microparticles recorded from the second heating.

Formulations	T_g_ (°C)	T_cc_ (°C) ^a^	ΔH_cc_ (J/g)	T_m_ (°C) ^b^	ΔH_m_ (J/g)
Ketorolac	43.72				
PLGA	42.79				
PLA	53.29	(110.67) 121.00	45.93	(167.67) 173.00	45.48
**Blank microparticles**					
PLGA (PS)	41.80				
PLGA/PLA (PS)	40.82, 53.95	100.67	5.48	171.33	8.28
PLA (PS)	51.90	(99.67) 106.67	11.49	172.67	15.00
PLGA (HSS)	45.70				
PLGA/PLA (HSS)	46.29, 58.72	117.33	20.82	174.00	20.06
PLA (HSS)	59.80	116.33	40.56	175.33	39.86
**Drug-loaded microparticles**					
PLGA (PS)	35.87				
PLGA/PLA (PS)	35.85, 48.01	(84.93) 100.00	8.26	(155.67) 163.00	12.11
PLA (PS)	48.73	(88.33) 99.67	19.52	(152.33) 162.67	21.82
PLGA (HSS)	34.94				
PLGA/PLA (HSS)	38.65, 48.95	(90.67) 104.33	12.70	(157.00) 165.67	12.58
PLA (HSS)	48.79	(89.67) 102.67	20.56	(153.67) 164.00	168.26

^a,b^ A number in parenthesis represents a small peak of the lower temperature of cold crystallization and melting, respectively.

## Data Availability

The data presented in this study are available on request from the corresponding author.

## Supplementary Materials

The following supporting information can be downloaded at: https://www.mdpi.com/article/10.3390/polym15020266/s1, Figure S1: DSC thermogram of ketorolac after the first and second heating; Table S1: Characteristics of blank microparticles prepared by PS and HSS techniques.
