# Supplementary material for: Ketorolac-Loaded PLGA-/PLA-Based Microparticles Stabilized by Hyaluronic Acid: Effects of Formulation Composition and Emulsification Technique on Particle Characteristics and Drug Release Behaviors
Source: Polymers (Basel). 2023 Jan 4;15(2):266. doi: 10.3390/polym15020266 (PMC9863719; doi:10.3390/polym15020266)
Supplement: Supplementary file 1 [file polymers-15-00266-s001.zip › polymers-2079023-supplementary.pdf]

Table S1. Characteristics of blank microparticles prepared by PS and HSS techniques.

Formulation	d (0.5)	d (0.9)	Span	ZP (mV)
PLGA (PS)	4.22 ± 0.05	13.99 ± 1.47	3.00 ± 0.31	-5.0 ± 0.4
PLGA/PLA (PS)	3.12 ± 0.04	21.62 ± 6.33	6.54 ± 2.03	-5.0 ± 0.4
PLA (PS)	7.72 ± 0.02	21.15 ± 0.31	2.47 ± 0.04	-3.8 ± 0.2
PLGA (HSS)	56.32 ± 0.20	89.32 ± 0.86	1.07 ± 0.01	-9.8 ± 0.8
PLGA/PLA (HSS)	82.81 ± 0.44	134.61 ± 1.49	1.20 ± 0.01	-5.4 ± 0.5
PLA (HSS)	97.44 ± 0.17	147.62 ± 0.72	1.11 ± 0.01	-4.7 ± 0.1

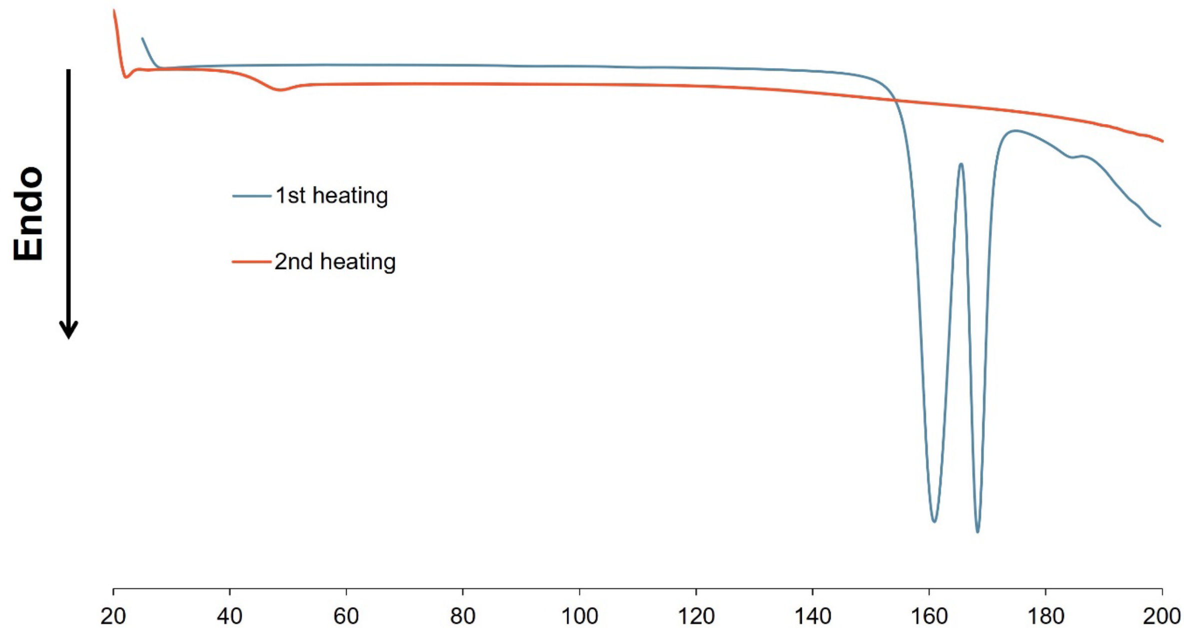

Figure S1. DSC thermogram of ketorolac after the first and second heating.
